# The Associations Between Gonadal Hormones and Serum Uric Acid Levels in Men and Postmenopausal Women With Diabetes

**DOI:** 10.3389/fendo.2020.00055

**Published:** 2020-02-20

**Authors:** Heng Wan, Kun Zhang, Yuying Wang, Yi Chen, Wen Zhang, Fangzhen Xia, Yunping Zhang, Ningjian Wang, Yingli Lu

**Affiliations:** ^1^Institute and Department of Endocrinology and Metabolism, Shanghai Ninth People's Hospital, Shanghai Jiao Tong University School of Medicine, Shanghai, China; ^2^Department of Endocrinology and Metabolism, The People's Hospital of Xiangyun, Shanghai, China

**Keywords:** uric acid, hyperuricemia, gonadal hormones, dehydroepiandrosterone, estradiol, testosterone

## Abstract

**Introduction:** In assessing the development of hyperuricemia in diabetic adults, the role of the sex steroid axis is underappreciated. Furthermore, dehydroepiandrosterone (DHEA) has been recommended as a nutritional supplement. However, is DHEA suitable for diabetic adults with hyperuricemia? This issue has received little attention.

**Aim:** The objective of this study was to investigate the associations between gonadal hormones and uric acid (UA) levels in diabetic adults, paying particular attention to the association between DHEA and UA levels.

**Methods:** We analyzed 4,426 participants out of 4,813 diabetic adults enrolled from seven communities in a cross-sectional survey conducted in 2018. Participants underwent several examinations, including assessments of anthropometric parameters, blood pressure, glucose, lipid profiles, UA, total testosterone (TT), estradiol (E2), the follicle-stimulating hormone (FSH), the luteinizing hormone (LH), and dehydroepiandrosterone (DHEA).

**Results:** Among men and compared with individuals in the first quartile, participants in the fourth quartile of TT and FSH had odds of hyperuricemia that were significantly decreased by so much as 48 and 34%, respectively (both *P* < 0.05). However, participants in the fourth quartile of DHEA had 79% increased odds of hyperuricemia (*P* < 0.05). Among postmenopausal women, participants in the fourth quartile of DHEA, TT, and LH had odds of hyperuricemia that were significantly increased by 155, 99, and 76%, respectively (all *P* < 0.05). These associations were adjusted for potential confounding factors.

**Conclusions:** Sex differences were found in the associations between gonadal hormones and UA levels in diabetic men and postmenopausal women, which should be monitored to prevent hyperuricemia when sex hormone treatment, especially DHEA, is administered. Further studies are needed.

## Introduction

Uric acid (UA) is the end product of purine metabolism in humans. In the past few decades, the prevalence of hyperuricemia has rapidly increased worldwide. A recent epidemiological survey reported that the age-adjusted prevalence of hyperuricemia was 8.02% in a total sample (6.87% in women and 8.57% in men) in China (1). A large number of observational studies have demonstrated that elevations in UA levels are independently associated with not only gout and urolithiasis (2), but also hypertension, type 2 diabetes mellitus (T2DM), metabolic syndrome, endothelial dysfunction, and all-cause mortality (3–6). On the basis of this evidence, studies have reported that hyperuricemia increases the risk of diabetic complications and all-cause mortality in diabetic adults (7, 8). Therefore, for the prevention of diabetic complications and mortality, it is important to explore the prevalence of hyperuricemia and its potential risk factors.

Gonadal hormone levels are linked to age and may have different impacts on the development of metabolic diseases in men and women (9). Although epidemiologic research has shown a steady growth of hyperuricemia with increasing age in all populations (10), in elderly men and postmenopausal women, the prevalence of hyperuricemia is different, which indicates that gonadal hormones may play a role. Our previous study, referred to as the Survey on Prevalence in East China for Metabolic Disease and Risk Factors (SPECT-China), reported that higher UA levels were associated with lower total testosterone (TT) and sex hormone-binding globulin (SHBG) levels in a total of 1,365 general men (age 55.5 ± 10.8 years) (11). However, as gonadal hormone levels are quite different between individuals with and without diabetes (12), it is vital to investigate the associations between UA levels and gonadal hormone levels among individuals with diabetes. One recent study showed that, among diabetic men, hypogonadism is related to higher UA levels (13), but few studies have simultaneously analyzed the associations between gonadal hormones and UA in men and postmenopausal women with diabetes.

In addition, dehydroepiandrosterone (DHEA) has been recommended as a nutritional supplement because of its protective effects on metabolism, such as its anti-diabetes, anti-obesity and anti-atherosclerosis effects (14). Further studies have indicated that a higher prevalence of diabetic complications was associated with lower DHEA (15, 16). The vascular protective effects of DHEA may result from the nitric oxide released from intact vascular endothelial cells being acutely physiologically increased by DHEA concentrations (17). Thus, an increasing number of people with diabetes are taking DHEA to prevent diabetic complications. However, little attention has been paid to the question of whether it is suitable for diabetic adults with hyperuricemia.

Thus, in this study of a large community-based sample, we aimed to analyze the relationship between UA levels and endogenous gonadal hormone levels, including DHEA, TT, follicle-stimulating hormone (FSH), luteinizing hormone (LH), and estradiol (E2), in men and postmenopausal women with diabetes; we especially focused on the association between UA and DHEA levels, highlighting the importance of using gonadal hormones as important biochemical markers in both clinical and basic studies on sex-specific mechanisms of hyperuricemia. Our findings may provide evidence for the prevention and treatment of hyperuricemia in patients with diabetes.

## Methods

### Study Design and Participants

**Figure 1:**
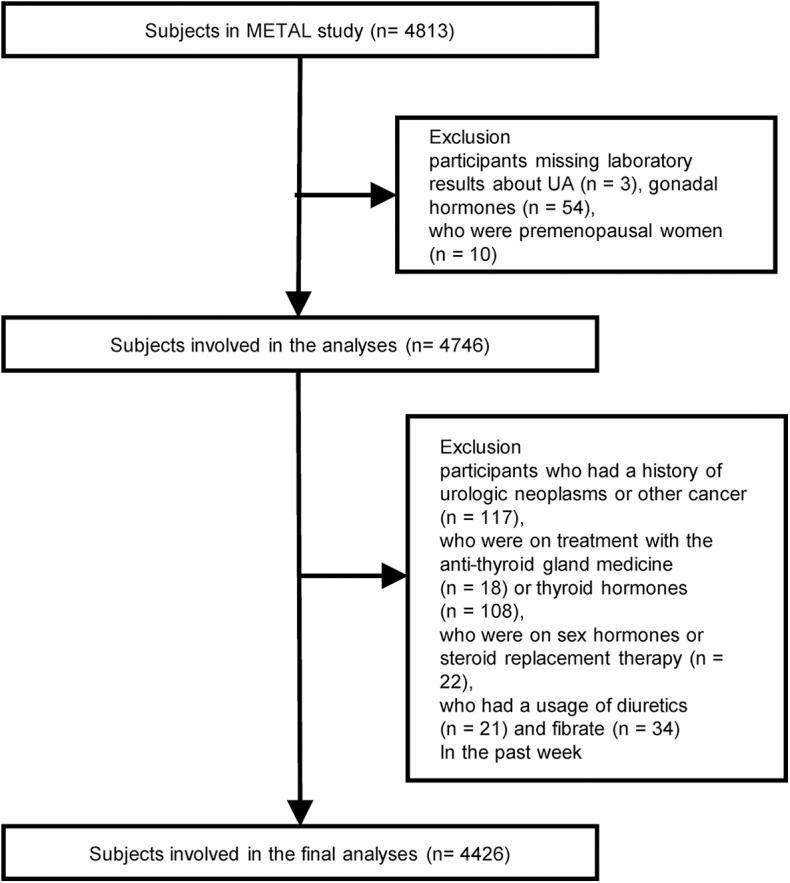
Flowchart of the sampling frame and participants selected from the METAL study.

We designed a cross-sectional study called the Environmental Pollutant Exposure and Metabolic Diseases in Shanghai (METAL study, www.chictr.org.cn, ChiCTR1800017573) to investigate the association between gonadal hormones and UA levels in Chinese adults with diabetes. The participants were enrolled from seven communities in Huangpu and Pudong District, Shanghai, China. Chinese citizens ≥ 18 years old who had lived in their current area for ≥ 6 months were included. Postmenopausal women were defined as subjects who reported that they had stopped menstruating for a minimum of 12 months and who were 55 years of age or older or those who had previous hysterectomy or oophorectomy; FSH ≥ 25.0 IU/L was required for all menopause criteria (according to the 2011 Stages of Gonadal Aging Workshop +10 recommendation, late perimenopausal state is characterized as FSH level ≥ 25 IU/L) (18). In August 2018, 4,813 subjects underwent an examination. We excluded participants who were missing laboratory results for UA and gonadal hormones, including TT, E2, LH, FSH, and DHEA (*n* = 57); who were premenopausal (*n* = 10); who had a history of urologic neoplasms or other cancer (*n* = 117); who were on treatment with the anti-thyroid gland medicine (*n* = 18) or thyroid hormones (*n* = 108), on sex hormones or steroid replacement therapy (*n* = 22) and who used diuretics or fibrates (*n* = 55) in the past week. Finally, 4,426 participants with diabetes were involved in the final analyses (Figure 1).

### Measurements

A questionnaire about sociodemographic characteristics, medical history, family history, and lifestyle factors, including smoking, alcohol consumption, and dietary habits, was administered during the interview, as in previous studies (19, 20). The same group of trained and experienced personnel from the SPECT-China study (21) conducted the interviews and clinical examinations, including inspections of weight, height, and blood pressure, according to a standard protocol. Current smoking was defined as having smoked at least 100 cigarettes in one's lifetime and currently smoking cigarettes (22). People who consumed alcohol at least once per day for at least 6 months continuously were defined as current alcohol consumers (23).

Blood samples were obtained between 6:00 and 9:00 a.m. after the participants fasted for at least 8 h. Blood was refrigerated immediately after phlebotomy, centrifuged within 2 h, and the serum was then aliquoted and frozen in a central laboratory.

TT, E2, FSH, and LH were measured by electrochemiluminescence immunoassay (Roche Cobas E601, Basel, Switzerland). DHEA and insulin were detected using the chemiluminescence method (Abbott i2000 SR, Chicago, USA). The minimal detectable limit for each gonadal hormone was as follows: 0.087 nmol/L (TT), 18.4 pmol/L (E2), 0.1 IU/L (FSH and LH), and 3.0 pg/mL (DHEA). UA, fasting plasma glucose (FPG), serum creatinine, triglyceride (TG), total cholesterol (TC), high-density lipoprotein (HDL), and low-density lipoprotein (LDL) were measured with a Beckman Coulter AU 680 (Brea, USA). Glycated hemoglobin (HbA1c) was measured by high-performance liquid chromatography (MQ-2000PT, Medconn, Shanghai, China). Samples with values below the minimal detectable limit were assigned a value midway between zero and the minimal detectable limit for the analyses (24). The interassay and intra-assay coefficients of variation were 8.33 and 6.25%, respectively, for TT, E2, FSH, and LH and 4.21 and 1.89%, respectively, for DHEA.

Hyperuricemia was defined as UA > 420 μmol/L (7 mg/dl) in both men and postmenopausal women (25). The presence of diabetes was determined when a previous diagnosis had been made by a healthcare professional, when FPG was ≥7.0 mmol/L, or when HbA1c was ≥6.5% according to the American Diabetes Association (26). Hypertension was indicated by systolic blood pressure ≥ 140 mmHg, diastolic blood pressure ≥ 90 mmHg, or self-reported previous diagnosis of hypertension by a physician. Dyslipidemia was defined as TC ≥ 6.22 mmol/L (240 mg/dL), TG ≥ 2.26 mmol/L (200 mg/dL), LDL ≥ 4.14 mmol/L (160 mg/dL), HDL < 1.04 mmol/L (40 mg/dL), or a self-reported previous diagnosis of hyperlipidemia by a physician according to the modified National Cholesterol Education Program-Adult Treatment Panel III. The estimated glomerular filtration rate (eGFR) was calculated according to the Chronic Kidney Disease Epidemiology Collaboration equation for “Asian origin” (27).

Weight (kilograms) and height (centimeters) were measured using a stadiometer and a vertical ruler while subjects wore light clothing without shoes. Body mass index (BMI) was calculated as weight in kilograms divided by height in meters squared.

### Statistical Analysis

Data analyses were performed using IBM SPSS Statistics, Version 22 (IBM Corporation, Armonk, NY, USA). A *P* < 0.05 indicated significance (two sided). Continuous variables are summarized as the mean ± SD or median (interquartile range), and categorical variables are summarized as percentages (%). Continuous variables were compared using Student's *t*-test. The Mann-Whitney U test was used for non-normally distributed continuous variables, and the Pearson χ^2^ test was used for dichotomous variables. Linear or logistic regression analysis was used to test for trends in the variable changes across the DHEA and TT quartiles, providing unadjusted *P*-values for trends.

The associations between serum UA and gonadal hormone level quartiles in adults with diabetes were assessed by multiple linear regression. Data are expressed as unstandardized coefficients (β) (95% confidence intervals). The model was adjusted for alcohol consumption status, smoking status, duration of diabetes, hypertension, dyslipidemia, BMI, HbA1c, eGFR (including age), and the usage of aspirin, losartan, and irbesartan.

The subjects were subsequently divided into normal and hyperuricemia groups. The odds ratios (ORs) and 95% confidence intervals (CIs) were calculated using logistic regression to determine the risk of hyperuricemia for each quartile of reproductive hormone levels by using the first quartile as the reference. The same model was adjusted separately.

Sensitivity analyses were performed. We conducted further subgroup analysis based on the subjects with or without hyperuricemia. We also calculated the associations between gonadal hormones and UA level in adults with diabetes after excluding ±3 SD values of UA levels by multiple linear regression. The same model was adjusted. In addition, to investigate whether the treatment for diabetes affects the association, we analyzed the associations between UA level or prevalence of hyperuricemia and gonadal hormone level quartiles after adjusting for the model, including alcohol consumption status, smoking status, duration of diabetes, hypertension, dyslipidemia, BMI, HbA1c, eGFR, the usage of aspirin, losartan, and irbesartan, and the usage of metformin, sulfonylurea or glinides, alpha-glucosidase inhibitor, dipeptidyl peptidase-4 (DPP-4) inhibitors, glucagon-like peptide-1 (GLP-1) analogs, thiazolidinediones, sodium-glucose cotransporter-2 (SGLT2) inhibitors, and insulin. A *P*-value (two sided) < 0.05 indicated significance.

## Results

### Characteristics of Participants With and Without Hyperuricemia

The characteristics of the study subjects with and without hyperuricemia are summarized in Table 1. A total of 4,426 subjects (2,164 men and 2,262 postmenopausal women) were involved in the final analyses. The mean age of the study population was 67.44 ± 8.77 years among men and 67.79 ± 7.75 years among postmenopausal women. The prevalence of hyperuricemia, hypertension, and dyslipidemia was 13.2% (16.9% in men and 9.8% in postmenopausal women), 78.8% (78.1% in men and 79.5% in postmenopausal women), and 61.9% (63.5% in men and 60.3% in postmenopausal women), respectively. Compared to those without hyperuricemia [HU (–)], men with hyperuricemia [HU (+)] had a significantly longer duration of diabetes; greater BMI; lower levels of FPG, HbA1c, HDL, eGFR, and TT; higher levels of TG and DHEA; and a higher prevalence of hypertension and dyslipidemia (*P* < 0.05). Compared to those without hyperuricemia [HU (–)], postmenopausal women with hyperuricemia [HU (+)] were older and had a significantly greater BMI; lower levels of HDL and eGFR; higher levels of TG, TT, FSH, LH, and DHEA; and a higher prevalence of hypertension and dyslipidemia (*P* < 0.05).

**Table 1:** General characteristics of subjects with and without hyperuricemia (HU).

**Characteristic**	**Men**			**Postmenopausal women**		
	**HU (–)**	**HU (+)**	***P* Value**	**HU (–)**	**HU (+)**	***P* Value**
*N*	1,798	366	–	2 041	221	–
Age, year	67.48 ± 8.49	67.22 ± 10.04	0.635	67.49 ± 7.61	70.45 ± 8.51	<0.001
Duration of diabetes, year	10 (4,15)	8 (3,15)	0.038	9 (3,15)	10 (3, 16.5)	0.563
Current smoking, %	36.8	33.8	0.309	2.5	2.3	0.844
Current alcohol use, %	39.4	42.1	0.347	5.7	3.2	0.122
BMI, kg/m^2^	24.85 ± 3.30	25.86 ± 3.30	<0.001	24.60 ± 3.63	26.75 ± 4.20	<0.001
FPG, mmol/L	7.96 ± 2.41	7.29 ± 1.95	<0.001	7.74 ± 2.44	7.69 ± 2.32	0.782
HbA1c, %	7.67 ± 1.46	7.17 ± 1.09	<0.001	7.41 ± 1.35	7.34 ± 1.22	0.450
TC, mmol/L	4.80 ± 1.08	4.88 ± 1.26	0.259	5.37 ± 1.21	5.45 ± 1.34	0.373
TG, mmol/L	1.38 (1.01, 1.99)	1.73 (1.26, 2.52)	<0.001	1.56 (1.13, 2.20)	2.00 (1.44, 2.99)	<0.001
HDL, mmol/L	1.12 ± 0.26	1.06 ± 0.23	<0.001	1.31 ± 0.30	1.17 ± 0.25	<0.001
LDL, mmol/L	2.99 ± 0.79	3.01 ± 0.81	0.764	3.29 ± 0.87	3.33 ± 0.90	0.538
UA, μmol/L	318.10 ± 58.34	473.62 ± 54.25	<0.001	294.67 ± 60.96	465.77 ± 47.22	<0.001
Hypertension, %	76.9	83.9	0.003	78.5	89.1	<0.001
Dyslipidemia, %	61.6	73.0	<0.001	58.6	76.9	<0.001
eGFR, ml/min per 1.73 m^2^	92.27 ± 15.84	81.33 ± 20.53	<0.001	93.74 ± 14.49	75.61 ± 21.81	<0.001
TT, nmol/L	14.28 (11.03, 18.37)	12.93 (9.74, 16.57)	<0.001	0.53 (0.29, 0.81)	0.60 (0.40, 0.99)	<0.001
E2, pmol/L	116.10 (91.57, 139.90)	121.75 (93.87, 145.85)	0.091	39.74 (9.18, 59.91)	37.76 (9.18, 50.67)	0.072
FSH, IU/L	9.57 (6.52, 14.39)	9.34 (5.88, 13.93)	0.867	53.25 (39.98, 68.42)	54.46 (40.41, 74.03)	0.007
LH, IU/L	7.21 (5.30, 9.87)	7.32 (5.34, 10.12)	0.829	24.69 (18.20, 31.67)	27.96 (21.23, 36.22)	<0.001
DHEA, pg/mL	171.35 (118.98, 236.40)	180.35 (119.90, 262.93)	0.011	117.10 (78.40, 162.95)	126.70 (86.90, 187.55)	0.006
**Medicine use**						
Losartan and irbesartan, %	7.1	10.2	0.042	6.7	10.6	0.034
Aspirin, %	6.7	7.9	0.367	3.7	5.0	0.357

*The data are summarized as the mean ± SD or median (interquartile range) for continuous variables or as a numerical proportion for categorical variables. P-values were calculated by Student's t-test, the Mann–Whitney U-test, or Pearson's χ^2^ test. P-values < 0.05 were considered significantly different. HU, hyperuricemia; BMI, body mass index; FPG, fasting plasma glucose; HbA1c, glycated hemoglobin; TC, total cholesterol; TG, triglyceride; HDL, high-density lipoprotein; LDL, low-density lipoprotein; UA, uric acid; eGFR, glomerular infiltration rate; TT, total testosterone; E2, estradiol; FSH, follicle-stimulating hormone; LH, luteinizing hormone; DHEA, dehydroepiandrosterone*.

### Metabolic Characteristics of Subjects by DHEA and TT Quartiles

The metabolic characteristics of subjects grouped by DHEA-level quartiles are shown in Table 2. Compared with the men in the lowest DHEA quartile, men in the highest quartile were younger, had a shorter duration of diabetes, had greater UA and eGFR levels, and had a higher prevalence of dyslipidemia (all *P* for trend < 0.05). Compared with the postmenopausal women in the lowest DHEA quartile, postmenopausal women in the highest quartile were younger, had a shorter duration of diabetes and had higher FPG, UA, and eGFR (all *P* for trend < 0.05). Supplementary Table 1 shows the metabolic characteristics of subjects grouped by TT level quartiles.

**Table 2:** Metabolic characteristics of subjects by DHEA quartiles.

**Characteristic**	**DHEA, pg/mL**				***P* for trend**
	**Q1**	**Q2**	**Q3**	**Q4**	
**Men**					
DHEA, pg/mL	≤ 119.15	119.16–172.85	172.86–240.70	>240.70	–
*N*	541	541	542	540	–
Age, year	71.84 ± 8.51	68.29 ± 7.69	66.36 ± 7.86	63.26 ± 8.73	<0.001
Duration of diabetes, year	10 (4,18)	8 (4,15)	10 (3,15)	8 (3,15)	0.001
BMI, kg/m^2^	25.14 ± 3.27	24.77 ± 3.23	24.94 ± 3.33	25.23 ± 3.44	0.505
FPG, mmol/L	7.76 ± 2.30	7.81 ± 2.33	7.89 ± 2.44	7.93 ± 2.33	0.195
HbA1c, %	7.62 ± 1.41	7.54 ± 1.39	7.64 ± 1.48	7.53 ± 1.38	0.558
TC, mmol/L	4.77 ± 1.19	4.80 ± 1.16	4.80 ± 1.02	4.89 ± 1.07	0.079
TG, mmol/L	1.44 (1.07, 1.99)	1.39 (1.03, 2.08)	1.40 (1.02, 2.03)	1.52 (1.08, 2.21)	0.353
HDL, mmol/L	1.09 ± 0.25	1.10 ± 0.25	1.12 ± 0.26	1.12 ± 0.25	0.090
LDL, mmol/L	2.98 ± 0.84	2.98 ± 0.82	3.00 ± 0.75	3.04 ± 0.75	0.171
UA, μmol/L	344.60 ± 87.02	337.71 ± 76.60	336.96 ± 82.11	358.36 ± 80.37	0.010
Hypertension, %	78.7	78.6	76.4	78.5	0.720
Dyslipidemia, %	67.7	66.0	60.3	60.2	0.002
eGFR, ml/min per 1.73 m^2^	86.62 ± 17.42	88.93 ± 16.90	92.32 ± 16.17	93.81 ± 17.50	<0.001
**Postmenopausal women**					
DHEA, pg/mL	≤ 79.20	79.03–118.05	118.06–164.40	>164.40	–
*N*	567	564	567	564	–
Age, yr	69.84 ± 7.66	68.41 ± 7.39	66.89 ± 7.53	65.98 ± 7.85	<0.001
Duration of diabetes, yr	9 (3,15)	10 (4,18)	8 (3,15)	8 (3,15)	0.133
BMI, kg/m^2^	24.73 ± 3.75	24.68 ± 3.74	24.94 ± 3.74	24.89 ± 3.75	0.300
FPG, mmol/L	7.51 ± 2.23	7.79 ± 2.60	7.67 ± 2.43	7.95 ± 2.42	0.009
HbA1c, %	7.40 ± 1.29	7.42 ± 1.40	7.36 ± 1.26	7.44 ± 1.38	0.790
TC, mmol/L	5.28 ± 1.19	5.38 ± 1.20	5.42 ± 1.26	5.42 ± 1.21	0.055
TG, mmol/L	1.58 (1.14, 2.24)	1.63 (1.16, 2.31)	1.58 (1.17, 2.16)	1.64 (1.13, 2.30)	0.357
HDL, mmol/L	1.30 ± 0.29	1.27 ± 0.30	1.30 ± 0.31	1.30 ± 0.30	0.571
LDL, mmol/L	3.23 ± 0.88	3.31 ± 0.85	3.32 ± 0.92	3.33 ± 0.85	0.055
UA, μmol/L	297.70 ± 77.70	307.79 ± 79.41	314.28 ± 76.33	325.84 ± 77.81	<0.001
Hypertension, %	80.8	78.0	79.0	80.5	0.968
Dyslipidemia, %	60.8	60.6	58.7	61.3	0.964
eGFR, ml/min per 1.73 m^2^	89.74 ± 17.48	92.15 ± 15.36	92.13 ± 15.67	93.86 ± 16.27	<0.001

*The data are summarized as the mean ± SD or median (interquartile range) for continuous variables or as a numerical proportion for categorical variables. P for trend was calculated by regression tests. BMI, body mass index; FPG, fasting plasma glucose; HbA1c, glycated hemoglobin; TC, total cholesterol; TG, triglyceride; HDL, high-density lipoprotein; LDL, low-density lipoprotein; UA, uric acid; eGFR, glomerular infiltration rate; DHEA, dehydroepiandrosterone*.

### Associations Between Gonadal Hormones and UA Levels

Table 3 shows the associations between gonadal hormones and UA levels in adults with diabetes. Compared with men in the first quartile, after adjusting for alcohol consumption status, smoking status, duration of diabetes, hypertension, dyslipidemia, BMI, HbA1c, eGFR, and the usage of aspirin, losartan, and irbesartan, men in the fourth quartile of TT and FSH had lower UA levels [(β −22.67, 95% CI −35.29, and −12.75) and (β −14.79, 95% CI −24.41, and −5.17), respectively] (both *P* < 0.05). The UA level also decreased with the increased TT and FSH quartiles (*P* for trend < 0.05). However, compared with men in the first quartile of DHEA, men in the fourth quartile had higher UA levels after adjusting for the same variables (β 23.27, 95% CI 13.93, and 32.62) (*P* < 0.05). The UA level also increased significantly with increasing DHEA quartiles (*P* for trend < 0.05). No significant associations between E2, LH, and UA levels were found among men. Among the postmenopausal women, after adjusting for the same variables, compared with the first quartile, individuals in the fourth quartile of DHEA, TT, and LH had higher UA levels [(β 34.70, 95% CI 26.26, and 43.15), (β 19.49, 95% CI 10.80, and 28.19) and (β 10.77, 95% CI 1.88, and 19.67), respectively] (all *P* < 0.05). The UA level also increased significantly with the increased DHEA, TT, and LH quartiles (all *P* for trend < 0.05), and no significant associations were found between E2, FSH, and UA among postmenopausal women.

**Table 3:** Associations between gonadal hormones quartiles and UA level.

	**Quartile 1**	**Quartile 2**	**Quartile 3**	**Quartile 4**	***P* for trend**	**1 SD increment in gonadal hormone**
**Men**						
DHEA	Ref.	−3.65 (−12.86, 5.57)	−1.37 (−10.69, 7.95)	23.27 (13.93, 32.62)	<0.001	11.40 (8.09, 14.72)
TT	Ref.	−10.92 (−20.23, −1.60)	−8.26 (−17.75, 1.23)	−22.67 (−32.59, −12.75)	<0.001	−8.10 (−11.60, −4.60)
E2	Ref.	−8.14 (−17.48, 1.20)	−7.02 (−16.40, 2.37)	−3.48 (−12.91, 5.95)	0.543	−1.56 (−5.05, 1.93)
LH	Ref.	−4.89 (−14.33, 4.56)	−5.11 (−14.63, 4.42)	−7.89 (−17.72, 1.95)	0.133	−5.45 (−9.04, −1.87)
FSH	Ref.	−9.37 (−18.74, 0.01)	−11.28 (−20.77, −1.78)	−14.79 (−24.41, −5.17)	0.003	−5.34 (−8.81, −1.88)
**Postmenopausal women**						
DHEA	Ref.	14.63 (6.23, 23.04)	19.10 (10.62, 27.58)	34.70 (26.26, 43.15)	<0.001	11.29 (8.32, 14.26)
TT	Ref.	12.16(3.72, 20.61)	11.57 (2.97, 20.17)	19.49 (10.80, 28.19)	<0.001	5.94 (2.89, 8.99)
E2	Ref.	7.18 (−2.46, 16.82)	2.52 (−5.28, 10.34)	−2.20 (−10.27, 5.87)	0.673	−0.05 (−3.18, 3.07)
LH	Ref.	−0.88 (−9.40, 7.65)	−0.49 (−8.98, 8.00)	10.77 (1.88, 19.67)	0.030	5.60 (2.33, 8.86)
FSH	Ref.	−0.37 (−8.94, 8.20)	−6.50 (−15.23, 2.23)	−1.57 (−10.69, 7.55)	0.444	−0.14 (−3.41, 3.13)

*Data are expressed as regression coefficients (95% CIs). Linear regression analysis was used. Each gonadal hormone was separately adjusted for confounding factors. The model was adjusted for alcohol consumption status, smoking status, duration of diabetes, hypertension, dyslipidemia, BMI, HbA1c, eGFR (including age) and the usage of aspirin, losartan, and irbesartan. UA, uric acid; BMI, body mass index; HbA1c, glycated hemoglobin; eGFR, estimated glomerular infiltration rate; TT, total testosterone; E2, estradiol; FSH, follicle-stimulating hormone; LH, luteinizing hormone; DHEA, dehydroepiandrosterone*.

### Associations Between Gonadal Hormone Levels and the Prevalence of Hyperuricemia

The results of the associations between gonadal hormone levels and the prevalence of hyperuricemia in adults with diabetes are shown in Table 4. Among men, after adjusting for alcohol consumption status, smoking status, duration of diabetes, hypertension, dyslipidemia, BMI, HbA1c, eGFR, and the usage of aspirin, losartan, and irbesartan, compared with the first quartile, participants in the fourth quartile of TT and FSH had odds of hyperuricemia that were significantly decreased by 48% (OR 0.52, 95% CI 0.35, and 0.77) and 34% (OR 0.66, 95% CI 0.45, and 0.95) (both *P* < 0.05). However, participants in the fourth quartile of DHEA had odds of hyperuricemia that were increased by 79% (OR 1.79, 95% CI 1.26, and 2.53; *P* < 0.05) after adjusting for the same variables. Hyperuricemia was not significantly associated with E2 or LH levels among men. Among postmenopausal women, after adjusting for the same variables, participants in the fourth quartile of DHEA, TT, and LH had odds of hyperuricemia that were significantly increased by 155% (OR 2.55, 95% CI 1.58, and 4.13), 99% (OR 1.99, 95% CI 1.18, and 3.33), and 76% (OR 1.76, 95% CI 1.05, and 2.94), respectively (all *P* < 0.05). No associations were found between E2 and FSH and the prevalence of hyperuricemia.

**Table 4:** Associations between gonadal hormone quartiles and the prevalence of HU.

	**Quartile1**	**Quartile 2**	**Quartile 3**	**Quartile 4**	***P* for trend**	**1 SD increment in gonadal hormone**
**Men**						
DHEA	Ref.	0.82 (0.57, 1.19)	0.87 (0.59, 1.28)	1.79 (1.26, 2.53)	0.001	1.29 (1.14, 1.46)
TT	Ref.	0.64 (0.45, 0.91)	0.72 (0.51, 1.03)	0.52 (0.35, 0.77)	0.003	0.79 (0.68, 0.92)
E2	Ref.	0.96 (0.66, 1.40)	1.00 (0.69, 1.45)	1.34 (0.94, 1.92)	0.097	1.07 (0.94, 1.20)
LH	Ref.	1.08 (0.75, 1.56)	0.88 (0.61, 1.28)	0.93 (0.63, 1.36)	0.497	0.93 (0.80, 1.07)
FSH	Ref.	0.66 (0.46, 0.95)	0.77 (0.54, 1.20)	0.66 (0.45, 0.95)	0.058	0.94 (0.82, 1.07)
**Postmenopausal women**						
DHEA	Ref.	1.49 (0.90, 2.48)	1.25 (0.74, 2.12)	2.55 (1.58, 4.13)	<0.001	1.34 (1.15, 1.56)
TT	Ref.	2.00 (1.19, 3.37)	1.62 (0.94, 2.80)	1.99 (1.18, 3.33)	0.043	1.17 (1.01, 1.36)
E2	Ref.	1.44 (0.84, 2.48)	1.40 (0.89, 2.18)	1.09 (0.70, 1.71)	0.630	1.00 (0.84, 1.18)
LH	Ref.	1.39 (0.83, 2.34)	1.26 (0.75, 2.11)	1.76 (1.05, 2.94)	0.051	1.22 (1.04, 1.44)
FSH	Ref.	1.26 (0.78, 2.04)	0.82 (0.49, 1.37)	1.05 (0.63, 1.74)	0.729	1.08 (0.91, 1.28)

*Data are expressed as odds ratios (95% CIs). Logistic regression analysis was used. Each gonadal hormone was separately adjusted for confounding factors. The model was adjusted for alcohol consumption status, smoking status, duration of diabetes, hypertension, dyslipidemia, BMI, HbA1c, eGFR (including age), and the usage of aspirin, losartan, and irbesartan. HU, hyperuricemia; BMI, body mass index; HbA1c, glycated hemoglobin; eGFR, glomerular infiltration rate; TT, total testosterone; E2, estradiol; FSH, follicle-stimulating hormone; LH, luteinizing hormone; DHEA, dehydroepiandrosterone*.

### Sensitivity Analyses

Among diabetic men without hyperuricemia, DHEA (positively) and TT (negatively) were still associated with UA levels; however, FSH was not significantly associated with UA levels. Among diabetic postmenopausal women without hyperuricemia, DHEA, and TT were still positively associated with UA levels; however, LH was not significantly associated with UA levels (Supplementary Table 2). Among diabetic men and postmenopausal women with hyperuricemia, no significant association was found between gonadal hormones (including DHEA, TT, LH, FSH, and E2) and UA levels (Supplementary Table 3). These associations were all adjusted for alcohol consumption status, smoking status, duration of diabetes, hypertension, dyslipidemia, BMI, HbA1c, eGFR, and the usage of aspirin, losartan, and irbesartan. The differences between the participants with and without hyperuricemia may be account for the number of the participants with hyperuricemia was too small.

To eliminate the effects of extreme uric acid levels on the results, we calculated the associations between gonadal hormones and UA levels after excluding ± 3 SD values of UA. Fifteen men and 12 postmenopausal women were excluded. The associations of TT and DHEA with UA levels in men and postmenopausal women remained consistent (Supplementary Table 4).

Among men, the percentage of participants taking metformin, sulfonylurea or glinides, alpha-glucosidase inhibitor, DPP-4 inhibitor, GLP-1 analogs, thiazolidinediones, SGLT2-inhibitor, and insulin was 41.1, 42.3, 27.7, 2.4, 0.1, 3.8, 0.1, and 19.0%, respectively. The percentage of participants without taking antidiabetic drugs was 8.3%. Among postmenopausal women, the percentage of participants taking metformin, sulfonylurea or glinides, alpha-glucosidase inhibitor, DPP-4 inhibitor, GLP-1 analogs, thiazolidinediones, SGLT2-inhibitor, and insulin was 42.8, 43.9, 27.9, 2.6, 0.1, 4.0, 0.1, and 16.2%, respectively. The percentage of participants without taking antidiabetic drugs was 9.5%. After adjusting for the further model including the usage of antidiabetic medicine, the associations between gonadal hormones quartiles and UA level (Supplementary Table 5) and the associations between gonadal hormone quartiles and the prevalence of hyperuricemia (Supplementary Table 6) remained as consistent as before.

## Discussion

In this study, among over 4,000 community-dwelling Chinese adults, we reported that DHEA (positively), TT (negatively), and FSH (negatively) were associated with UA levels in men, and DHEA, TT, and LH were all positively associated with UA levels in postmenopausal women after adjusting for possible confounders. To the best of our knowledge, this study is the first to evaluate the associations between gonadal hormones and UA levels in a large population including men and postmenopausal women with diabetes simultaneously. The sex differences in the association between gonadal hormones and UA levels may indicate sex-specific mechanisms of hyperuricemia and the function of UA, using gonadal hormones as biochemical markers. More importantly, our results showed that DHEA was associated with UA levels in both men and postmenopausal women with diabetes, which should draw public attention to the question of whether DHEA should be used in diabetic adults with hyperuricemia.

DHEA, a biomarker of hypothalamic-pituitary-adrenal axis activity (28), is a steroid hormone that has several effects on metabolism, such as anti-diabetes, anti-obesity, and anti-atherosclerosis effects (29, 30); DHEA has also been available as a health food supplement in the USA. However, few studies have investigated the adverse effects of DHEA. As far as we know, only one study, the participants in which were recruited from a single hospital, reported that DHEA was positively associated with UA (13). In our study, a positive and significant association between DHEA and UA was observed in both men and postmenopausal women.

We suspected that the possible mechanism is that DHEA may increase sodium reabsorption and reduce water removal, as it has been reported that DHEA could activate mineralocorticoid receptor and inhibit glucocorticoid receptor (31), which would lead to serum UA concentration increases by reduced renal UA excretion (13). However, the specific mechanism is still not clear. Further research is needed to clarify the correlation and mechanism between DHEA and UA.

In the present study, gonadal hormone levels were differently associated with UA levels between men and postmenopausal women. The UA level increased with decreasing testosterone levels in men; however, the UA level increased with increasing testosterone levels in postmenopausal women. A previous study found an inverse association between testosterone levels and UA levels among men with and without T2DM separately (11, 13). These results are consistent with our findings. However, in postmenopausal women, testosterone administration was reported to cause a definite increase in plasma UA levels (32). Furthermore, some reports have shown that testosterone treatment for female to male gender reassignment leads to increased serum UA concentrations (33, 34). These findings are similar to ours. As few studies have reported the relationship between endogenous testosterone and UA in postmenopausal women, our study may supplement the knowledge on this relationship.

The association between E2 and UA is controversial. Although postmenopausal status has been recognized as a significant determinant of higher serum UA concentrations in women in the general population (35), Krishnan considered that aging, not menopause, was associated with a higher prevalence of hyperuricemia among older women (36). However, a reduction in serum UA has been observed after accepting hormone replacement therapy in postmenopausal women with hyperuricemia (37). Interestingly, Jung, J. H. evaluated the effects of different types of hormone therapy on serum UA levels and attributed the findings to the effects of progestogen, rather than E2 (38), which is similar to the result of our study showing that E2 is not associated with UA in postmenopausal women. We also observed no association between E2 and UA in men, which is consistent with the results of a previous study (13).

The associations between FSH, LH, and UA levels are controversial. One previous study reported that both men and women with gout had lower FSH and LH levels (39). Two other studies have shown that LH and FSH decreased with elevated UA levels in men (11, 13). We also observed that FSH was negatively associated with UA among men with diabetes and that LH had a positive association with UA among postmenopausal women with diabetes. The different results may be caused by factors associated with the different populations. Further studies are needed to reveal this relationship.

Although our study had some strengths, including novelty, the inclusion of a relatively large sample of community-dwelling participants and strong quality control, there were also some limitations. First, this is a cross-sectional study; thus, causal relationships between gonadal hormones and UA cannot be confirmed. Second, the participants in this study were all diabetic adults; thus, the results may not be generalizable to the entire population. Third, we did not measure the SHBG and free testosterone levels in the present study while considering the cost, which may limit the investigation on the relationship between testosterone and UA in patients with diabetes. Further cohort or randomized controlled studies with larger sample sizes are still required.

## Conclusions

DHEA (positively), TT (negatively), and FSH (negatively) were associated with UA levels among men with diabetes, and DHEA, TT, and LH were all positively associated with UA levels in postmenopausal women with diabetes after adjusting for possible confounders. This may provide a new clinical understanding and assist in the treatment of hyperuricemia in diabetic patients. Furthermore, our findings suggest that the level of UA be monitored to prevent hyperuricemia when DHEA is administered in diabetic adults, although the definite effect and potential pathogenic effect of DHEA on UA levels both need further investigation.

## Data Availability Statement

The raw data supporting the conclusions of this article will be made available by the authors, without undue reservation, to any qualified researcher.

## Ethics Statement

The study protocol was approved by the Ethics Committee of Shanghai Ninth People's Hospital, Shanghai Jiao Tong University School of Medicine. The study protocol conformed to the ethical guidelines of the 1975 Declaration of Helsinki as reflected in a priori approval by the appropriate institutional review committee. Written informed consent was obtained from all participants included in the study.

## Author Contributions

YL and NW designed the study. HW, YW, YC, WZ, and FX conducted the research. HW, KZ, and YZ analyzed the data. HW wrote the manuscript. The final manuscript was read and approved by all authors.

### Conflict of Interest

The authors declare that the research was conducted in the absence of any commercial or financial relationships that could be construed as a potential conflict of interest.

## Abbreviations

UA: Uric acid
HU: hyperuricemia
T2DM: Type 2 diabetes mellitus
TT: Total testosterone
E2: Estradiol
FSH: Follicle-stimulating hormone
LH: Luteinizing hormone
DHEA: Dehydroepiandrosterone
SHBG: sex hormone-binding globulin
FPG: Fasting plasma glucose
TG: Triglyceride
TC: Total cholesterol
HDL: High-density lipoprotein
LDL: Low-density lipoprotein
HbA1c: Glycated hemoglobin
eGFR: Estimated glomerular filtration rate
BMI: Body mass index
OR: Odds ratio
CI: Confidence interval
DPP-4: dipeptidyl peptidase-4
GLP-1: glucagon-like peptide-1
and SGLT2: sodium-glucose cotransporter-2.
