# Supplementary material for: The Associations Between Gonadal Hormones and Serum Uric Acid Levels in Men and Postmenopausal Women With Diabetes
Source: Front Endocrinol (Lausanne). 2020 Feb 20;11:55. doi: 10.3389/fendo.2020.00055 (PMC7044188; doi:10.3389/fendo.2020.00055)
Supplement: Supplementary file 1 [file Data_Sheet_1.docx]

Supplementary table 1. Metabolic characteristics of subjects by TT quartiles

| Characteristic | TT, nmol/L | | | | *P* for trend |
| --- | --- | --- | --- | --- | --- |
|  | Q1 | Q2 | Q3 | Q4 |  |
| Men |  | | | | |
| *N* | 545 | 537 | 541 | 541 | - |
| TT, nmol/L | ≤10.82 | 10.83-14.12 | 14.13-18.01 | >18.01 | - |
| Age, yr | 67.34±9.63 | 66.58±9.11 | 67.37±8.11 | 68.45±8.06 | 0.014 |
| Duration of diabetes, yr | 8 (3,15) | 9 (4,15) | 8 (3,15) | 10 (5,18) | 0.079 |
| BMI, kg/m^2^ | 26.27±3.35 | 25.62±3.33 | 24.81±2.90 | 23.36±2.96 | <0.001 |
| FPG, mmol/L | 8.31±2.57 | 7.80±2.28 | 7.70±2.21 | 7.56±2.25 | <0.001 |
| HbA1c, % | 7.70±1.38 | 7.60±1.35 | 7.53±1.40 | 7.51±1.52 | 0.017 |
| TC, mmol/L | 4.79±1.26 | 4.75±1.10 | 4.86±1.03 | 4.87±1.04 | 0.108 |
| TG, mmol/L | 1.71 (1.24, 2.62) | 1.49 (1.09, 2.14) | 1.40 (1.08, 1.98) | 1.16 (0.86, 1.67) | <0.001 |
| HDL, mmol/L | 1.05±0.23 | 1.07±0.23 | 1.11±0.24 | 1.21±0.29 | <0.001 |
| LDL, mmol/L | 2.94±0.84 | 2.97±0.80 | 3.05±0.77 | 3.03±0.76 | 0.024 |
| UA, μmol/L | 358.97±85.89 | 343.79±80.98 | 348.21±77.81 | 326.51±79.95 | <0.001 |
| Hypertension, % | 84.2 | 80.1 | 77.1 | 70.8 | <0.001 |
| Dyslipidemia, % | 73.0 | 70.2 | 62.7 | 48.2 | <0.001 |
| eGFR, ml/min per 1.73 m^2^ | 91.02±18.00 | 91.67±16.16 | 90.00±16.65 | 89.00±17.89 | 0.020 |
| Postmenopausal women |  |  |  |  |  |
| *N* | 567 | 564 | 567 | 564 | - |
| TT, pg/mL | ≤0.30 | 0.31-0.54 | 0.55-0.83 | >0.83 | - |
| Age, yr | 69.84±7.66 | 68.41±7.39 | 66.89±7.53 | 65.98±7.85 | <0.001 |
| Duration of diabetes, yr | 9 (3,15) | 10 (4,18) | 8 (3,15) | 8 (3,15) | 0.307 |
| BMI, kg/m^2^ | 24.73±3.75 | 24.68±3.74 | 24.94±3.74 | 24.89±3.75 | <0.001 |
| FPG, mmol/L | 7.51±2.23 | 7.79±2.6 | 7.67±2.43 | 7.95±2.42 | <0.001 |
| HbA1c, % | 7.4±1.3 | 7.42±1.4 | 7.36±1.26 | 7.44±1.38 | <0.001 |
| TC, mmol/L | 5.28±1.2 | 5.38±1.2 | 5.42±1.26 | 5.42±1.21 | 0.951 |
| TG, mmol/L | 1.58 (1.14, 2.24) | 1.63 (1.16, 2.32) | 1.58 (1.17, 2.16) | 1.64 (1.13, 2.30) | 0.180 |
| HDL, mmol/L | 1.30±0.29 | 1.27±0.3 | 1.30±0.31 | 1.30±0.3 | 0.101 |
| LDL, mmol/L | 3.23±0.88 | 3.31±0.85 | 3.32±0.92 | 3.33±0.85 | 0.591 |
| UA, μmol/L | 297.7±77.7 | 307.79±79.41 | 314.28±76.33 | 325.84±77.81 | <0.001 |
| Hypertension, % | 80.8 | 78.0 | 79.0 | 80.5 | 0.104 |
| Dyslipidemia, % | 60.8 | 60.6 | 58.7 | 61.3 | 0.047 |
| eGFR, ml/min per 1.73 m^2^ | 89.74±17.48 | 92.15±15.36 | 92.13±15.67 | 93.86±16.27 | 0.023 |

The data are summarized as the mean± SD or median (interquartile range) for continuous variables or as a numerical proportion for categorical variables. *P* for trend was calculated by regression tests.

BMI, body mass index; FPG, fasting plasma glucose; HbA1c, glycated hemoglobin; TC, total cholesterol; TG, triglyceride; HDL, high-density lipoprotein; LDL, low-density lipoprotein; UA, uric acid; eGFR, glomerular infiltration rate; TT, total testosterone.

Supplementary table 2. Associations between gonadal hormones and UA in diabetic adults without hyperuricemia

|  | Quartile1 | Quartile 2 | Quartile 3 | | Quartile 4 | | *P* for trend | 1SD increment of gonadal hormone |
| --- | --- | --- | --- | --- | --- | --- | --- | --- |
| Men |  |  |  | |  | |  |  |
| DHEA | Ref. | 2.69 (-4.86, 10.24) | 2.95 (-4.61, 10.50) | | 18.45 (10.74, 26.17) | | <0.001 | 8.54 (5.80, 11.27) |
| TT | Ref. | -6.27 (-13.83, 1.28) | -1.74 (-9.45, 5.97) | | -15.08 (-23.12, -7.03) | | 0.002 | -5.80 (-8.64, -2.96) |
| E2 | Ref. | -9.20 (-16.78, -1.62) | -9.22 (-16.81, -1.62) | | -9.67 (-17.33, -2.02) | | 0.019 | -2.86 (-5.70, -0.02) |
| LH | Ref. | -8.89 (-16.56, -1.20) | -3.70 (-11.39, 3.99) | | -7.77 (-15.65, 0.11) | | 0.149 | -3.63 (-6.53, -0.73) |
| FSH | Ref. | -0.14 (-7.79, 7.50) | -2.57 (-10.25, 5.11) | | -6.34 (-14.17, 1.49) | | 0.090 | -3.50 (-6.32, 0.68) |
| Postmenopausal women | | | |  | |  |  |  |
| DHEA | Ref. | 9.20 (1.81, 16.58) | 17.43 (9.99, 24.87) | | 26.72 (19.29, 34.14) | | <0.001 | 9.12 (6.52, 11.72) |
| TT | Ref. | 8.62 (1.23, 16.00) | 6.18 (-1.33, 13.69) | | 14.18 (6.59, 21.77) | | 0.001 | 4.60 (1.93, 7.27) |
| E2 | Ref. | 4.60 (-4.19, 13.38) | 0.40 (-6.39, 7.18) | | -2.77 (-9.76, 4.22) | | 0.460 | 0.12 (-2.61, 2.84) |
| LH | Ref. | -1.90 (-9.34, 5.55) | -2.46 (-9.90, 4.98) | | 4.10 (-3.61, 11.80) | | 0.382 | 1.79 (-1.04, 4.62) |
| FSH | Ref. | -1.93 (-9.40, 5.54) | -4.23 (-11.83, 3.36) | | -1.76 (-9.65, 6.14) | | 0.535 | -2.06 (-4.87, 0.76) |

1798 men and 2041 postmenopausal women without hyperuricemia were included.

Data are expressed as regression coefficients (95%CI). Linear Regression analysis was used respectively in men and postmenopausal women. Each of gonadal hormones was separately adjusted for confounding factors. The model was adjusted for drinking status, smoking status, duration of diabetes, hypertension, dyslipidemia, BMI, HbA1c, eGFR (including age) and the usage of aspirin, Losartan and Irbesartan.

UA, uric acid; BMI, body mass index; HbA1c, glycated hemoglobin; eGFR, estimated glomerular infiltration rate; TT, total testosterone; E2, estradiol; FSH, follicle-stimulating hormone; LH, luteinizing hormone; DHEA, dehydroepiandrosterone.

Supplementary table 3. Associations between gonadal hormones and UA in diabetic adults with hyperuricemia

|  | Quartile1 | Quartile 2 | Quartile 3 | | Quartile 4 | | *P* for trend | 1SD increment of gonadal hormone |
| --- | --- | --- | --- | --- | --- | --- | --- | --- |
| Men |  |  |  | |  | |  |  |
| DHEA | Ref. | -17.49 (-34.43, -0.54) | -14.02 (-31.11, 3.08) | | -10.62 (-27.55, 6.32) | | 0.260 | -2.69 (-8.75, 3.38) |
| TT | Ref. | 2.19 (-14.80, 19.17) | -1.66 (-18.88, 15.55) | | -9.24 (-27.11, 8.63) | | 0.288 | -0.34 (-6.69, 6.01) |
| E2 | Ref. | -8.89 (-25.81, 8.03) | -4.01 (-21.32, 13.30) | | -1.18 (-18.55, 16.20) | | 0.051 | -2.40 (-8.72, 3.92) |
| LH | Ref. | 2.07 (-15.28, 19.43) | 0.42 (-17.26, 18.11) | | 3.66 (-15.44, 22.75) | | 0.301 | -3.40 (-10.24, 3.44) |
| FSH | Ref. | -20.52 (-37.18, -3.87) | -26.35 (-43.61, -9.10) | | -15.03 (-32.54, 2.49) | | 0.071 | -4.47 (-10.79, 1.84) |
| Postmenopausal women | | | |  | |  |  |  |
| DHEA | Ref. | 17.60 (-0.40, 35.61) | 10.89 (-6.82, 28.60) | | 6.57 (-10.63, 23.78) | | 0.692 | 0.45 (-5.57, 6.47) |
| TT | Ref. | 6.00 (-11.23, 23.23) | 10.92 (-6.56, 28.39) | | 7.02 (-10.52, 24.55) | | 0.336 | 2.23 (-3.86, 8.32) |
| E2 | Ref. | -10.24 (-27.60, 7.12) | -4.10 (-21.64, 13.43) | | -19.62 (-37.95, -1.29) | | 0.080 | -2.96 (-9.79, 3.86) |
| LH | Ref. | -3.92 (-21.46, 13.62) | -10.73 (-28.12, 6.66) | | 16.32 (-2.64, 35.29) | | 0.254 | 4.59 (-2.25, 11.43) |
| FSH | Ref. | -12.68 (-30.37, 5.02) | -15.70 (-30.13, 2.73) | | -2.36 (-22.14, 17.43) | | 0.709 | 2.66 (-4.41, 9.73) |

366 men and 221 postmenopausal women with hyperuricemia were included.

Data are expressed as regression coefficients (95%CI). Linear Regression analysis was used respectively in men and postmenopausal women. Each of gonadal hormones was separately adjusted for confounding factors. The model was adjusted for drinking status, smoking status, duration of diabetes, hypertension, dyslipidemia, BMI, HbA1c, eGFR (including age) and the usage of aspirin, Losartan and Irbesartan.

UA, uric acid; BMI, body mass index; HbA1c, glycated hemoglobin; eGFR, estimated glomerular infiltration rate; TT, total testosterone; E2, estradiol; FSH, follicle-stimulating hormone; LH, luteinizing hormone; DHEA, dehydroepiandrosterone.

Supplementary table 4. Associations between gonadal hormones and UA in adults with diabetes after excluding ±3 SD values of UA level

|  | Quartile1 | Quartile 2 | Quartile 3 | Quartile 4 | *P* for trend | 1SD increment of gonadal hormone |
| --- | --- | --- | --- | --- | --- | --- |
| Men |  |  |  |  |  |  |
| TT | Ref. | -10.56 (-19.61, -1.50) | -6.57(-15.81, 2.67) | -20.92 (-30.55, -11.28) | <0.001 | -7.79 (-11.19, -4.38) |
| E2 | Ref. | -6.14 (-15.22, 2.94) | -8.12 (-17.26, 1.03) | -5.69 (-14.86, 3.48) | 0.198 | -2.48 (-5.88, 0.92) |
| LH | Ref. | -6.56 (-15.78, 2.67) | -6.46 (-15.71, 2.79) | -9.82 (-19.37, -0.28) | 0.056 | -5.76 (-9.25, -2.27) |
| FSH | Ref. | -8.35 (-17.48, 0.79) | -9.20 (-18.43, 0.03) | -14.52 (-23.91, -5.13) | 0.004 | -5.09 (-8.47, -1.71) |
| DHEA | Ref. | -1.13 (-10.13, 7.87) | 0.13 (-8.94, 9.20) | 25.66 (16.54, 34.78) | <0.001 | 12.51 (9.28, 15.75) |
| Postmenopausal women | | |  |  |  |  |
| TT | Ref. | 11.85 (3.59, 20.10) | 6.85 (-1.49, 15.19) | 17.12 (8.70, 25.48) | 0.001 | 5.12 (2.14, 8.10) |
| E2 | Ref. | 8.09 (-1.39, 17.58) | 3.41 (-4.23, 11.05) | -2.26 (-10.12, 5.60) | 0.691 | -0.09 (-3.14, 2.96) |
| LH | Ref. | 0.33 (-7.97, 8.62) | 0.31 (-7.98, 8.60) | 12.00 (3.32, 20.68) | 0.014 | 4.79 (1.62, 7.96) |
| FSH | Ref. | 0.79 (-7.54, 9.12) | -6.21 (-14.75, 2.33) | -0.63 (-9.53, 8.26) | 0.520 | -0.64 (-3.82, 2.55) |
| DHEA | Ref. | 13.52 (5.28, 21.76) | 16.08 (7.81, 24.34) | 33.69 (25.44, 41.94) | <0.001 | 10.79 (7.91, 13.68) |

Data are expressed as regression coefficients (95%CI). Linear Regression analysis was used.

Each of gonadal hormones was separately adjusted for confounding factors. The model was adjusted for drinking status, smoking status, duration of diabetes, hypertension, dyslipidemia, BMI, HbA1c, eGFR (including age) and the usage of aspirin, Losartan and Irbesartan.

UA, uric acid; BMI, body mass index; HbA1c, glycated hemoglobin; eGFR, glomerular infiltration rate; TT, total testosterone; E2, estradiol; FSH, follicle-stimulating hormone; LH, luteinizing hormone; DHEA, dehydroepiandrosterone.

Supplementary table 5. Associations between gonadal hormones quartiles and UA level after adjusting for the further model including the usage of antidiabetic medicine

|  | Quartile 1 | Quartile 2 | Quartile 3 | | Quartile 4 | | *P* for trend | 1 SD increment in gonadal hormone |
| --- | --- | --- | --- | --- | --- | --- | --- | --- |
| Men |  |  |  | |  | |  |  |
| DHEA | Ref. | -3.51 (-13.64, 6.61) | -1.67 (-11.92, 8.57) | | 20.63 (10.33, 30.93) | | <0.001 | 10.08 (6.39, 13.77) |
| TT | Ref. | -12.75 (-22.94, -2.57) | -11.57 (-22.09, -1.06) | | -24.30 (-35.18, -13.42) | | <0.001 | -8.48 (-12.34, -4.63) |
| E2 | Ref. | -7.94 (-18.23, 2.35) | -6.25 (-16.64, 4.14) | | -1.99 (-12.45, 8.47) | | 0.817 | -1.89 (-5.86, 2.08) |
| LH | Ref. | -5.42 (-15.96, 5.13) | -6.17 (-16.77, 4.43) | | -10.96 (-21.87, 0.51) | | 0.088 | -7.03 (-10.91, 1.15) |
| FSH | Ref. | -16.89 (-27.31, -6.48) | -14.28 (-24.81, -3.75) | | -22.24 (-32.85, -11.63) | | <0.001 | -6.89 (-10.64, -3.15) |
| Postmenopausal women | | | |  | |  |  |  |
| DHEA | Ref. | 14.95 (5.85, 24.06) | 19.47 (10.27, 28.67) | | 33.56 (24.36, 42.76) | | <0.001 | 10.50 (7.28, 13.72) |
| TT | Ref. | 13.32 (4.19, 22.43) | 11.13 (1.78, 20.47) | | 20.51 (11.05, 29.97) | | <0.001 | 5.65 (2.29, 9.02) |
| E2 | Ref. | 7.21 (-3.37, 17.78) | 1.18 (-7.35, 9.72) | | -1.51 (-10.24, 7.22) | | 0.722 | -0.81 (-4.60, 2.98) |
| LH | Ref. | -1.68 (-11.01, 7.64) | -4.37 (-13.69, 4.95) | | 6.98 (2.75, 16.71) | | 0.036 | 3.87 (0.30, 7.45) |
| FSH | Ref. | -1.94 (-11.35, 7.47) | -9.01 (-18.58, 0.56) | | -2.59 (-12.62, 7.43) | | 0.347 | -0.71 (-4.29, -2.86) |

Data are expressed as regression coefficients (95% CIs). Linear regression analysis was used.

Each gonadal hormone was separately adjusted for confounding factors. The model was adjusted for alcohol consumption status, smoking status, duration of diabetes, hypertension, dyslipidemia, BMI, HbA1c, eGFR, the usage of aspirin, losartan and irbesartan and the usage of metformin, sulfonylurea or glinides, alpha-glucosidase inhibitor, DPP-4 inhibitors, GLP-1 analogues, thiazolidinediones, SGLT2 inhibitors and insulin.

UA, uric acid; BMI, body mass index; HbA1c, glycated hemoglobin; eGFR, estimated glomerular infiltration rate; TT, total testosterone; E2, estradiol; FSH, follicle-stimulating hormone; LH, luteinizing hormone; DHEA, dehydroepiandrosterone; DPP-4: dipeptidyl peptidase-4; GLP-1: glucagon-like peptide-1; SGLT2: sodium-glucose cotransporter-2.

Supplementary table 6. Associations between gonadal hormone quartiles and the prevalence of HU after adjusting for the further model including the usage of antidiabetic medicine

|  | Quartile1 | Quartile 2 | Quartile 3 | Quartile 4 | *P* for trend | 1 SD increment in  gonadal hormone |
| --- | --- | --- | --- | --- | --- | --- |
| Men |  |  |  |  |  |  |
| DHEA | Ref. | 0.73 (0.48, 1.10) | 0.79 (0.51, 1.20) | 1.61 (1.10, 2.36) | 0.015 | 1.25 (1.09, 1.44) |
| TT | Ref. | 0.59 (0.40, 0.87) | 0.64 (0.43, 0.94) | 0.49 (0.32, 0.75) | 0.002 | 0.76 (0.64, 0.90) |
| E2 | Ref. | 0.79 (0.52, 1.19) | 0.84 (0.56, 1.27) | 1.11 (0.75, 1.65) | 0.512 | 0.96 (0.82, 1.13) |
| LH | Ref. | 1.00 (0.66, 1.51) | 0.79 (0.52, 1.20) | 0.77 (0.50, 1.18) | 0.143 | 0.86 (0.72, 1.02) |
| FSH | Ref. | 0.57 (0.38, 0.85) | 0.72 (0.48, 1.07) | 0.54 (0.36, 0.82) | 0.015 | 0.89 (0.76, 0.99) |
| Postmenopausal women | | |  |  |  |  |
| DHEA | Ref. | 1.42 (0.81, 2.49) | 1.18 (0.67, 2.10) | 2.49 (1.46, 4.24) | 0.002 | 1.34 (1.13, 1.58) |
| TT | Ref. | 1.49 (0.84, 2.63) | 1.41 (0.78, 2.53) | 1.76 (1.01, 3.07) | 0.049 | 1.15 (1.01, 1.37) |
| E2 | Ref. | 1.41 (0.78, 2.56) | 1.26 (0.77, 2.06) | 1.00 (0.61, 1.63) | 0.284 | 0.95 (0.77, 1.18) |
| LH | Ref. | 1.16 (0.65, 2.05) | 1.06 (0.60, 1.88) | 1.44 (1.02, 2.52) | 0.045 | 1.15 (1.06, 1.38) |
| FSH | Ref. | 1.10 (0.64, 1.89) | 0.76 (0.43, 1.35) | 0.96 (0.55, 1.67) | 0.599 | 1.05 (0.87, 1.27) |

Data are expressed as odds ratios (95% CIs). Logistic regression analysis was used. Each gonadal hormone was separately adjusted for confounding factors. The model was adjusted for alcohol consumption status, smoking status, duration of diabetes, hypertension, dyslipidemia, BMI, HbA1c, eGFR, the usage of aspirin, losartan and irbesartan and the usage of metformin, sulfonylurea or glinides, alpha-glucosidase inhibitor, DPP-4 inhibitors, GLP-1 analogues, thiazolidinediones, SGLT2 inhibitors and insulin.

HU, hyperuricemia; BMI, body mass index; HbA1c, glycated hemoglobin; eGFR, glomerular infiltration rate; TT, total testosterone; E2, estradiol; FSH, follicle-stimulating hormone; LH, luteinizing hormone; DHEA, dehydroepiandrosterone; DPP-4: dipeptidyl peptidase-4; GLP-1: glucagon-like peptide-1; SGLT2: sodium-glucose cotransporter-2.
